# Difficulties with prescribed opioids: a cross-sectional survey of primary care patients in England, United Kingdom

**DOI:** 10.1097/PR9.0000000000001246

**Published:** 2025-02-25

**Authors:** Louise E. Wilson, Roger D. Knaggs, Anthony J. Avery, Tracey Thornley, José Moss, Reham M. Baamer, Matthew J. Boyd

**Affiliations:** a Division of Pharmacy Practice and Policy, School of Pharmacy, University of Nottingham, Nottingham, United Kingdom; b Centre for Academic Primary Care, School of Medicine, University of Nottingham, Nottingham, United Kingdom; c Boots UK, Nottingham, United Kingdom; d Department of Pharmacy Practice, Faculty of Pharmacy, King Abdulaziz University, Jeddah, Saudi Arabia

**Keywords:** Opioids, Pain, Primary care, Cross-sectional survey

## Abstract

**Introduction:**

Knowledge of the potential for harm and adverse effects from long-term opioid medicines has led to reduced prescribing in recent years. However, the number of patients receiving opioid prescriptions from primary care in England remains substantial and understanding the difficulties patients experience with these medicines is important for the provision of care.

**Objective:**

To understand the difficulties experienced by patients prescribed opioids for noncancer pain in primary care.

**Methods:**

A cross-sectional postal survey of adults prescribed an opioid medicine for noncancer pain over a period of ≥3 months from 10 general practices (n = 3077) in the East Midlands was conducted using self-completed questionnaires. Sociodemographic, pain, and opioid use information was gathered to characterise the study population. An adapted version of the 15-item Prescribed Opioids Difficulties Scale was used to assess the problems and concerns attributed to using prescribed opioids, from the time opioids were first prescribed.

**Results:**

Questionnaires were received from 619 respondents (response rate = 20.1%), of whom 59.8% were female, and the median age was 64 years. Four in 5 (79.8%) had experienced at least one opioid problem or concern from the Prescribed Opioids Difficulties Scale, and one in 5 (22.8%) had experienced 8 or more. The most frequently reported difficulty was needing to increase the dose of opioids to get the same effect (46.0%). Chi-square analysis with odds ratio found respondents were 2.6 times more likely to report ≥4 difficulties if they had higher pain intensity and interference (95% CI: 1.85–3.77, *P* < 0.001).

**Conclusion:**

Opioid difficulties were common in this population and this study provides evidence for regular monitoring of problems after prescribing.

## Abstract 2

Supplemental Digital Content is Available in the Text.

## 1. Introduction

Knowledge about the potential for harm and adverse effects from long-term use of opioid medicines, alongside limited benefits in the treatment of chronic noncancer pain (CNCP), has advanced over the past 20 years.^12,17,22,25,35^ Side effects such as constipation, nausea, dizziness, sedation, vomiting, and pruritus are known to occur frequently.^25^ Harm from problems related to opioid tolerance, dependence, and addiction is well documented, but the extent of these problems is less certain, particularly in the case of dependence.^20,22^ Reported levels of prescription opioid use disorder have been found to be very low in the United Kingdom, but are likely to be an underestimate because of a lack of diagnosis and documentation in patient records.^18^ This measure also fails to identify problems that fall below the threshold for opioid use disorder.

In recent years, countries with high levels of opioid prescribing have updated clinical guidelines to avoid or limit the use of opioids in many painful conditions, especially in CNCP.^13,21,46,51^ This change is reflected within guidance for health care practitioners in England, United Kingdom, produced by the National Institute for Health and Care Excellence (NICE).^37–41,43^ The United Kingdom National Health Service (NHS) requires patient registration with one general practice physician and uses a shared patient record system.^4^ This structure may have limited increases in opioid use in comparison with countries where patients have access to any medical practice, such as in the United States. Despite this, the United Kingdom has still experienced historical increases in opioid prescribing,^19,29^ followed by a period of gradual decline from 2016.^5,50^ In the absence of alternative medicines, the use of opioids for many types of pain remains substantial, with prescriptions for codeine accounting for the largest proportion of opioid prescribing in primary care.^29^ Several national initiatives and clinical interventions have been developed in the United Kingdom in recent years to support opioid dose reductions and/or discontinuation in CNCP.^3,8,47,48^ However, it is likely that many patients will continue to be prescribed some form of opioid medicine for the foreseeable future, and therefore require support to manage side effects and problems. Understanding the experiences of patients using these medicines, therefore, continues to be important for the provision of associated care.

The Prescribed Opioids Difficulties Scale (PODS) is a survey tool developed to assess recent issues associated with long-term opioid use for CNCP from the patient's perspective.^11^ Scale items examine problems and concerns attributed to opioids, including issues related to tolerance, dependence, and addiction. The survey has potential for wider use to assess opioid-related difficulties across the entire time opioids are prescribed. The Prescribed Opioids Difficulties Scale has been used in several studies in different countries^14,26,30,33,36^; however, to our knowledge, it has not previously been used in the United Kingdom. The main aim of this research was to assess the pain, opioid use, and difficulties experienced by patients prescribed opioids for noncancer pain, in a real-world setting, using an adapted version of the PODS.

## 2. Methods

The study design was a cross-sectional postal survey of adults prescribed an opioid medicine for noncancer pain by their general practice. Ethical approval was given by the United Kingdom Health Research Authority (ref 21/SC/0105).

### 2.1. Study population

Ten primary care general practices were recruited via the East Midlands Clinical Research Network, England, United Kingdom.^6^ The sampling frame included all adults aged 18 years and older, prescribed any opioid or opioid/paracetamol combination analgesic for noncancer pain over a period of ≥3 months (defined as ≥2 opioid prescriptions issued in the previous 3 months with a minimum of one opioid prescription in month 1, and one opioid prescription in month 3). Eligible patients were identified through an electronic search of the practice clinical system, followed by GP screening to check for exclusions. Patients with a terminal illness, unable to understand English, or vulnerable and deemed inappropriate to contact were excluded. Questionnaires were mailed, via CFH Docmail,^2^ with a letter from the practice, a letter from the research team, a participant information sheet, and a freepost returns envelope. A text message reminder to complete the questionnaire was sent 2 weeks after mailing to all eligible patients where the practice held a mobile telephone number. Questionnaires were mailed from the first practice in October 2021 and from the last practice in June 2022. Questionnaire returns were accepted until 31st July 2022.

Recruitment via general practices was considered the most appropriate method for identifying a sample of patients recently prescribed an opioid pain medicine. The majority of opioid prescriptions are issued from this setting, with 5.6 million adults in England (13% of the population) dispensed an opioid prescription for noncancer pain in 2017/18.^50^ The duration of opioid prescribing was chosen to give an adequate period of use on which participants could report their experience, not to determine long-term use. In this study, general practices completed all recruitment processes and the clinical information of eligible patients was not accessible to the research team. A sample size target of 600 patients was set to achieve a 4% margin of error, with a 95% confidence level, in descriptive statistics and detect any associations between categorical variables. A conservative 20% response estimate was used to recruit a sufficient number of practices from which 3000 eligible patients could be identified.

### 2.2. Experiences of prescribed opioids questionnaire

The survey collected information relating to prescribed opioids including participant sociodemographics, pain and opioid use, and difficulties experienced with opioid medicines (Supplemental Appendix 1, http://links.lww.com/PR9/A283). Survey questions used preexisting scales and nonscaled items developed from the literature and expertise within the research team. The survey was pretested with a PPI representative.

#### 2.2.1. Sociodemographic information

Data were collected for age, sex, ethnic group, and current employment status. Each participant was also assigned to an Index of Multiple Deprivation (IMD) quintile, determined by the profile of patients registered at the general practice from which they were recruited to the study.^7^

#### 2.2.2. Pain and prescribed opioid use

Participants were asked to complete a brief pain assessment using the Pain, Enjoyment of life, and General activity (PEG) scale.^32^ The PEG scale is composed of 3 numerical rating scales (range 0–10), assessing pain intensity and interference. Participants were also asked how long they had been using prescribed opioids for pain, on how many days they had used an opioid in the past 2 weeks, how helpful opioids have been in relieving pain over the past month, and whether opioids had relieved their pain as much as expected.

#### 2.2.3. Difficulties with opioid pain medicines

An adapted version of the PODS was used to assess the difficulties participants attributed to using prescribed opioids. The Prescribed Opioids Difficulties Scale is a validated scale, developed in the United States, composed of 8 items relating to psychosocial problems and 7 items relating to control concerns.^11^ The original version of the scale assesses current or recent difficulties to determine a problems and concerns score. We wanted to assess difficulties over the entire time opioids were prescribed; therefore, PODS scale items were not time-limited and, with the exception of one item, response options were presented as a 5-point agreement Likert scale.^34^

### 2.3. Data analysis

Responses were entered manually with an accuracy check on 10% of entries by a second member of the research team. IBM SPSS version 27 software program was used to conduct subsequent statistical analyses.

Pain, Enjoyment of life, and General activity scores were determined for each respondent by calculating the average score of the 3 scale items and grouping the results into low (0–3), medium (4–6), or high (7–10) scores, following the common categorisation of the Numerical Pain Rating Scale (NRPS).^1^ Days of opioid use in the past 2 weeks were grouped into less than 7, 7 to 13, and 14 (daily use). Values greater than 14 were treated as missing data.

Problems and concerns scores were calculated for each respondent by dichotomising each PODS item response into agreement/nonagreement. All questions scored 1 for agreement and 0 for nonagreement. One item, which asked about the impact of side effects, did not use a Likert agreement scale. For this the responses “no side effects” and “a little bothersome” scored 0, and “moderately”, “very,” and “extremely bothersome” scored 1. Total scores were subsequently grouped into “low” (0–3), “medium” (4–7), and “high” (8–15). Scoring and grouping reflected the original scoring of the scale as far as possible.^11^

Chi-square tests for independence were conducted to determine associations between respondent characteristics and how helpful they found opioids in relieving pain over the past month.^23^ Further chi-square tests were conducted between respondent characteristics and the problems and concerns score generated from the PODS questions. Odds ratios (OR) with 95% confidence intervals (CIs) were calculated for significant associations between dichotomous variables to give an indication of the strength of the association. A Bonferroni adjustment was made to the significance value to account for the multiple tests performed.^9^

## 3. Results

### 3.1. Demographics

General practices in the study had a combined list size of 101,774 patients (as reported on the date of electronic search), from which 3077 (3.0%) potential participants were identified as eligible to take part. A total of 619 questionnaires were received by the study team, giving a response rate of 20.1%. Demographic characteristics of respondents are presented in Table 1, with 59.8% categorised female and a median (IQR) age of 64 years (55–74 years). Just over half (50.7%) were retired and 59.9% were registered at a practice with a patient population within the 2 most deprived IMD quintiles.

**Table 1:** Demographic characteristics of respondents.

Characteristic	Classification	n	%
Sex (n = 610)	Female	365	59.8
	Male	241	39.5
	Other	4	0.7
Age (y) (n = 611)	18–29	4	0.7
	30–39	23	3.8
	40–49	54	8.8
	50–59	130	21.3
	60–69	179	29.3
	70–79	148	24.2
	80+	73	11.9
Ethnic background (n = 601)	Asian/Asian British	8	1.3
	Black/African/Caribbean/Black British	13	2.2
	Mixed/multiple ethnic groups	13	2.2
	White	567	94.3
Employment status* (n = 603)	Employed	126	20.9
	A student	3	0.5
	Retired	306	50.7
	A homemaker	15	2.5
	Unable to work	163	27.0
	Out of work and seeking		
	Opportunities	6	1.0
IMD quintile of participant GP practice† (n = 619)	1 (most deprived)	226	36.5
	2	145	23.4
	3	116	18.7
	4	74	12.0
	5 (least deprived)	58	9.4

* In some instances, respondents selected more than one answer.

† Respondents assigned to IMD quintile of GP Practice population on date of database search as published at https://fingertips.phe.org.uk/profile/general-practice.

IMD, Index of Multiple Deprivation.

### 3.2. Pain and prescribed opioid use

Pain intensity and interference (PEG) scores are shown in Table 2. Two-thirds of respondents (67.5%) had a calculated PEG score of between 7 and 10, indicating high pain intensity and interference. The proportion of high, medium, and low scores were similar within each of the 3 domains.

**Table 2:** PEG 3-item pain scale scores.

Scale component (range 0–10)	Low score (0–3)		Medium score (4–6)		High score (7–10)	
	n	%	n	%	n	%
Pain intensity (P) (n = 618)	27	4.4	188	30.4	403	65.2
Interference with enjoyment of life (E) (n = 616)	58	9.4	130	21.1	428	69.5
Interference with general activity (G) (n = 616)	52	8.4	149	24.2	415	67.4
Average (PEG) (n = 613)	36	5.9	163	26.6	414	67.5

Table 3 summarises the reported use of prescribed opioids by respondents. Nearly two-thirds (63.6%) had used prescribed opioid pain medicines for more than 3 years, with 82.5% reporting daily use in the past 2 weeks. Just over two-fifths (41.0%) found opioids very or extremely helpful in relieving their pain over the past month, with half (51.2%) reporting that opioids had probably or definitely relieved pain as much as they expected over the same time.

**Table 3:** Prescribed opioid use.

Characteristic	Classification	n	%
Duration of opioid use (n = 615)	3–6 mo	61	9.9
	7–12 mo	28	4.6
	1–2 y	54	8.8
	2–3 y	81	13.2
	>3 y	391	63.6
Opioid use in past 2 wk (n = 601)	<7 d	31	5.2
	7–13 d	74	12.3
	14 d	496	82.5
How helpful found opioids in relieving pain over the past month ? (n = 615)	Not at all helpful	13	2.1
	A little helpful	83	13.5
	Moderately helpful	267	43.4
	Very helpful	188	30.6
	Extremely helpful	64	10.4
Have opioids relieved pain as much as expected over the past month ? (n = 615)	Definitely not	47	7.6
	Probably not	134	21.8
	Might or might not	119	19.3
	Probably yes	217	35.3
	Definitely yes	98	15.9

### 3.3. Prescribed Opioids Difficulties Scale

Percentage responses to PODS items are shown in Table 4 (psychosocial problems) and Table 5 (control concerns). Problems with the highest levels of agreement from respondents were feeling slowed down, sluggish, or sedated (45.2%), and feeling sleepy or less alert when doing something where they needed to be alert (38.2%). One in 8 respondents (12.3%) considered any side effects of opioid pain medicines to be very or extremely bothersome.

**Table 4:** Percentage response for opioid problems.

	Strongly disagree		Disagree		Neutral		Agree		Strongly agree	
	n	%	n	%	n	%	n	%	n	%
There have been times when opioid pain medicines have caused me to lose interest in my usual activities (n = 610)	88	14.4	188	30.8	139	22.8	150	24.6	45	7.4
There have been times when opioid pain medicines have caused me to have trouble concentrating or remembering (n = 612)	78	12.7	195	31.9	142	23.2	155	25.3	42	6.9
There have been times when opioid pain medicines have caused me to feel slowed down, sluggish, or sedated (n = 613)	59	9.6	153	25.0	124	20.2	221	36.1	56	9.1
There have been times when opioid pain medicines have caused me to feel depressed, down, or anxious (n = 612)	108	17.6	191	31.2	147	24.0	123	20.1	43	7.0
There have been times when the side effects of opioid pain medicines have interfered with my work, family, or social responsibilities (n = 612)	106	17.3	180	29.4	142	23.2	141	23.0	43	7.0
There have been times when opioid pain medicines have made it hard for me to think clearly (n = 606)	94	15.5	209	34.5	139	22.9	137	22.6	27	4.5
There have been times when opioid pain medicines have made me sleepy or less alert when I was doing something where I needed to be alert (n = 604)	73	12.1	169	28.0	131	21.7	187	31.0	44	7.3

	No side effects			A little bothersome			Moderately bothersome			Very bothersome			Extremely bothersome		
	n		%	n		%	n		%	n		%	n		%
Considering any side effects of opioid pain medicines you may have experienced, how bothersome were these side effects ? (n = 602)	218		36.2	176		29.2	134		22.3	50		8.3	24		4.0

**Table 5:** Percentage response for opioid concerns.

	Strongly disagree		Disagree		Neutral		Agree		Strongly agree	
	n	%	n	%	n	%	n	%	n	%
There have been times when I have been preoccupied with or thought constantly about my opioid pain medicines (n = 602)	163	27.1	211	35.0	119	19.8	87	14.5	22	3.7
There have been times when I have felt that I could not control how much or how often I needed opioid pain medicines (n = 605)	173	28.6	216	35.7	98	16.2	90	14.9	28	4.6
There have been times when I have needed to increase the dose of my opioid pain medicines to get the same effect (n = 605)	93	15.4	133	22.0	101	16.7	230	38.0	48	7.9
There have been times when I have worried that I might be dependent on or addicted to opioid pain medicines (n = 611)	119	19.5	172	28.2	102	16.7	167	27.3	51	8.3
There have been times when I have wanted to stop my opioid pain medicines or to cut down on the amount that I use (n = 607)	88	14.5	195	32.1	135	22.2	148	24.4	41	6.8
There have been times when opioid pain medicines have caused me to have problems with family, friends, or coworkers (n = 606)	233	38.4	243	40.1	78	12.9	41	6.8	11	1.8
There have been times when family or friends have thought that I may be dependent on or addicted to opioid pain medicines (n = 608)	232	38.2	203	33.4	85	14.0	73	12.0	15	2.5

Concerns with the highest levels of agreement from respondents included needing to increase the dose to get the same effect (46.0%) and being worried that they might be dependent on or addicted to opioid pain medicines (35.7%). The level of agreement with other concerns was more variable, but notably, 31.1% of respondents had wanted to stop or cut down the amount used, and one in 12 (8.6%) reported opioid pain medicines had caused problems with family, friends, or coworkers.

**Figure 1.**
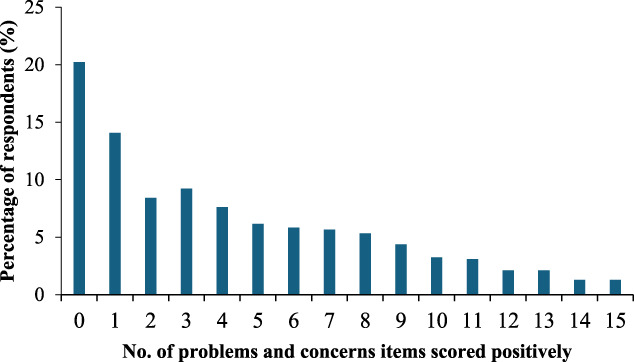
Distribution of problems and concerns scores.

The distribution of problems and concerns scores is presented in Figure 1. Four in 5 respondents (79.8%) had experienced at least one problem or concern attributed to opioid pain medicines, with almost half (48.1%) experiencing 4 or more. More than one in 5 respondents (22.8%) reported difficulties resulting in a high score (≥8 problems or concerns).

Associations between respondent characteristics and how helpful they found opioids in relieving pain over the past month are shown in Table 6. No significant associations were seen with sex, age, duration of opioid use, or daily opioid use. A significant association was seen with PEG score where those who reported high pain intensity and interference (high PEG score) found opioids less helpful than those with a lower score (*P* < 0.01).

**Table 6:** Associations between respondent characteristics and how helpful they found opioids in relieving pain over the past month.

Characteristic	Not at all/A little/Moderately helpful		Very/Extremely helpful		*P* *
	n	%	n	%	Chi-square
Sex (n = 602)					
Female	214	59.1	148	40.9	0.996
Male	141	58.8	99	41.3	
Age (n = 607)					
<50 y	48	59.3	33	40.7	
≥50 y	310	58.9	216	41.1	1.000
Duration of opioid use (n = 613)					
≤3 y	130	58.3	93	41.7	0.888
>3 y	231	59.2	159	40.8	
Daily opioid use (n = 599)					
Yes	298	60.1	198	39.9	0.252
No	55	53.4	48	46.6	
PEG score (n = 610)					
Low (0–3)	17	47.2	19	52.8	0.003†
Medium (4–6)	81	49.7	82	50.3	
High (7–10)	261	63.5	150	36.5	

† Significance reduced to *P* < 0.01 via Bonferroni adjustment.

* With Yates' continuity correction for all 2 × 2 tables.

PEG, pain intensity, interference with enjoyment, and interference with general activity.

Associations between respondent characteristics and problems and concerns score are shown in Table 7. No significant associations were seen with sex or duration of opioid use. Significant associations were seen with age, PEG score, and how helpful respondents found opioids in relieving pain over the past month (*P* < 0.01). The odds of a respondent reporting a medium to high problems and concerns score were 2.03 times higher when aged younger than 50 years, 2.18 times higher when they found opioids less helpful in relieving pain, and 2.64 times higher when they had a high PEG score. Approximately one-third of respondents had experienced ≥4 problems or concerns attributed to opioid pain medicines, while also reporting limited pain relief and/or high pain intensity and interference.

**Table 7:** Associations between respondent characteristics and problems and concerns score.

Characteristic	Problems and concerns				P* Chi-square	OR	95% CI
	Med-high score (4–15)		Low score (0–3)				
	n	%	n	%			
Sex (n = 605)							
Female	161	44.1	204	55.9	0.032	—	
Male	128	53.3	123	46.7			
Age (n = 610)							
<50 y	51	63.0	30	37.0	0.005†	2.03	1.25–3.29
≥50 y	241	45.6	288	54.4			
Duration of opioid use (n = 614)							
≤3 y	102	45.5	122	54.5	0.357	—	
>3 y	194	49.7	196	50.3			
PEG score (n = 612)							
High (7–10)	232	56.2	181	43.8	<0.001†	2.64	1.85–3.77
Low to medium (0–6)	65	32.7	134	67.3			
How helpful found opioids in relieving pain ? (n = 614)							
Not at all/a little/moderately	203	56.1	159	43.9	<0.001†	2.18	1.57–3.04
Very/extremely	93	36.9	159	63.1			

* With Yates' continuity correction.

† Reduced to *P* < 0.01 via Bonferroni adjustment.

PEG, pain intensity, interference with enjoyment, and interference with general activity.

## 4. Discussion

In this population of primary care patients, almost 80 percent reported at least one problem or concern attributed to prescribed opioids and over 20 percent reported 8 or more, highlighting the frequent occurrence of difficulties. Almost all reported opioids were helpful in relieving pain to some extent, but more than half reported they were only moderately, or a little helpful, and only half reported opioids had definitely or probably relieved pain as much as they expected in the past month. To our knowledge, this is the first study to assess opioid difficulties using the PODS for the entire duration of prescribing, providing valuable new insights into the problems and concerns of patients from their perspective.

Problems related to drowsiness or sedation were the most frequently reported, experienced by approximately 45% of respondents. This finding was consistent with other literature indicating these side effects are observed in 20% to 60% of patients using opioids.^16^ The impact of these side effects on daily life was highlighted by a similar proportion of respondents reporting drowsiness affecting their ability to be alert at times when they needed to be. Approximately 30% of respondents also reported opioids affecting concentration and memory, or the ability to think clearly, demonstrating further impact on daily activities and function. However, the proportion of respondents reporting side effects as very or extremely bothersome was lower at 12.3%, offering an insight into patient perceptions of these issues.

Opioid concerns relating to tolerance, dependence, or addiction were the most frequently reported. Almost half (46.0%) reported needing to increase the dose to get the same effect. This is interesting when considered in relation to current NICE guidance for medicines associated with dependence, published during the data collection period of this study.^42^ Guidance recommends prescribers should avoid automatically increasing the dose if the response is not sustained because reduced benefit is rarely due to pharmacological tolerance and the risk of harmful prescribing is increased. Nearly a third (31.1%) reported times when they wanted to stop or cut down the amount of opioid used. More respondents were worried they might be dependent on, or addicted to, opioids (35.7%) than reported not being able to control how much or how often they needed them (19.5%) or had been preoccupied with or thought constantly about them (18.1%). A previous survey in the United Kingdom and Ireland of people using codeine identified 17.1% of respondents as dependent using the Severity of Dependence Scale,^31^ a similar proportion to the dependence-related behaviours observed in this research. The levels of agreement we identified were higher than those reported in previous research using the PODS but followed a similar pattern of frequency across questions.^11,28,52^ This difference may be in part because difficulties were reported for the entire time opioids were prescribed.

Four in 5 respondents had used opioids every day for the past 2 weeks. Guidance on opioid use from the United Kingdom advises some patients may experience good pain relief when opioids are used intermittently.^24^ However, in this study, we did not find an association between nondaily use and how helpful respondents found opioids in relieving pain. A significant association was identified between pain intensity and interference (PEG score) and how helpful respondents found opioids in relieving their pain. High PEG scores were associated with finding opioids less helpful compared with low and medium PEG scores. Further analysis of the individual PEG domains (Supplemental Fig. 1, http://links.lww.com/PR9/A284) revealed the strongest association occurred between pain intensity and helpfulness. This study did not measure respondents experience of pain both with and without opioids. Therefore, we were unable to determine if patients with a high PEG score are less likely to respond to opioids, or if PEG scores remain high because opioids are not effective in treating pain in some patients. Approximately one in 6 respondents who found opioids very or extremely helpful reported a low to medium PEG score. However, more than a third of respondents with a high PEG score reported opioids were very or extremely useful in relieving their pain. This finding may be helpful in understanding why it can be difficult for patients to agree to opioid reductions even when the effect on pain intensity is limited. Previous studies in the United States have found no relationship between perceived helpfulness and pain severity or physical function scores, although these involved specific patient groups or conditions where opioids were expected to be less useful.^10,26^ Systematic reviews of opioid use have reported short-term efficacy in some patient groups and weak evidence for clinically significant pain relief in long-term use, with lower certainty for strong opioids.^15,27,44,45^ Many studies within these reviews also show a significant proportion of people taking opioids report high pain intensity. However, as highlighted above, there remains a gap in understanding the experience of pain when treated with opioids. The large level of reported helpfulness observed in this study was perhaps unexpected but may be explained by several factors. This study did not directly assess reductions in pain intensity, only patient perceptions of how helpful they found opioids in relieving pain over the past month. Patient perceptions of helpfulness may therefore have included very small changes in pain or improvements in issues associated with pain, such as sleep. Patients may also have had concerns about their opioid prescription being withdrawn, and worsening pain, if they reported opioids as less helpful. Where patients describe opioids as helpful, understanding the components of this is important to guide monitoring and support for these medicines.

Several studies have shown opioids are prescribed more frequently to female patients and this was observed in the respondent demographics of this research.^12,29,35^ However, we did not identify any further association between sex and the experience of opioid difficulties. Younger respondents, those who found opioids less helpful in relieving pain, and those reporting higher pain intensity and interference were associated with a greater number of problems and concerns. Previous research conducted in the United Kingdom has shown an association between younger age and opioid dependence,^18,31^ and our study indicates this association may be extended to other opioid difficulties.

### 4.1. Strengths and limitations

Although primary care practices recruited to this study were located within one geographical region, the sample is representative of people prescribed opioids for noncancer pain in England, over a period of at least 3 months, with respect to age, sex, and ethnicity.^35,50^ The study design enabled all eligible patients prescribed opioids at each participating practice to be identified and invited to take part, and a range of practices were recruited to the study with respect to socioeconomic factors. The survey response rate was modest at 20.1% but was consistent with the conservative estimate used in the study design and within the typical range for postal surveys.^49^ Nonresponse to individual questions was low, ranging from 0.2% to 2.9%, with no systematic omissions.

We were unable to determine if the sample characteristics of responders differed from nonresponders because of the ethical requirement for practice-held information about eligible patients to remain confidential. For the same reason, data on the type of opioid prescribed to participants were not available to the research team, and participants were not asked to provide this information. We recognise that having a general idea about the type of opioid prescribed could have been useful as harms are known to increase with higher morphine equivalent dose.^12^ Our aim was to assess the extent of difficulties in a real-world population, and it would have been difficult to accurately associate the opioid type and amount used with the point at which difficulties occurred. The proportion of respondents prescribed opioids for more than 3 years in the sample was larger than reported figures for England.^50^ There was potential for recruitment bias towards patients who had experienced difficulties with opioids and for whom pain had a greater impact on daily life. Recall bias may also have occurred because of the length of time some respondents had been prescribed opioids. It is also possible that the cause of difficulties experienced may not always have been opioids or solely because of opioids. Considering study strengths and limitations, we believe the results may be cautiously generalised.

## 5. Conclusion

This research identified opioid difficulties were frequently experienced by patients prescribed opioid pain medicines and regular monitoring of problems after prescribing may be needed. These findings can be used to inform future work to identify pain diagnoses, comorbidities, and prescribing indicators associated with opioid difficulties. Our findings also highlight opportunities and challenges for reducing opioid prescribing. We identified a substantial proportion of patients who have wanted to stop or cut down their opioid use and/or experienced multiple difficulties with little perceived benefit. These patients may respond more positively towards opioid reductions. However, a considerable proportion of patients in this study reported opioids were helpful in relieving pain, irrespective of the extent of difficulties experienced or the current intensity of pain. Persuading patients to reduce opioid use in these circumstances is more challenging. A person-centred approach to pain management is essential as individual experiences, expectations, and perceived benefits of using opioid medicines can vary significantly.

## Disclosures

A. J. Avery is National Clinical Director for Prescribing for NHS England. The remaining authors have no conflict of interest to declare.

## Appendix A. Supplemental digital content

Supplemental digital content associated with this article can be found online at http://links.lww.com/PR9/A283, and http://links.lww.com/PR9/A284.

## Supplementary Material

SUPPLEMENTARY MATERIAL
