# Supplementary material for: Difficulties with prescribed opioids: a cross-sectional survey of primary care patients in England, United Kingdom
Source: Pain Rep. 2025 Feb 25;10(2):e1246. doi: 10.1097/PR9.0000000000001246 (PMC11864306; doi:10.1097/PR9.0000000000001246)
Supplement: SUPPLEMENTARY MATERIAL [file painreports-10-e1246-s001.pdf]

Appendix 1 Questionnaire

University of Nottingham
UK | CHINA | MALAYSIA

Part 1: About your pain

Q1 What part(s) of the body is your pain? **Tick ALL that apply**

- All over the body ☐
- Head ☐
- Face ☐
- Neck or shoulder(s) ☐
- Arm(s) ☐
- Hand(s) ☐
- Chest ☐
- Stomach or abdomen ☐
- Back ☐
- Hip(s) ☐
- Knee(s) ☐
- Leg(s) ☐
- Feet ☐
- Other ☐

For **other** please tell us where:

Q2 What number best describes your **pain on average** in the past week?
Circle ONE number only

No pain	0	1	2	3	4	5	6	7	8	9	10	Pain as bad as you can imagine

---

Q3 What number best describes how, during the past week, pain has interfered with your **enjoyment of life? Circle ONE number only**

Does not interfere	0	1	2	3	4	5	6	7	8	9	10	Completely interferes

Q4 What number best describes how, during the past week, pain has interfered with your **general activity? Circle ONE number only**

Does not interfere	0	1	2	3	4	5	6	7	8	9	10	Completely interferes

Q5 How long have you been taking opioid pain medicines? **Tick ONE answer that fits best**

Note: If you have used opioid medicines for a different pain problem in the past, please tell us how long you have been using opioids for your current pain problem.

- 3 to 6 months ☐
- 7 to 12 months ☐
- 1 to 2 years ☐
- 2 to 3 years ☐
- More than 3 years ☐

Q6 In the past **TWO weeks**, on how many days have you taken an opioid pain medicine? **Give a number between 0 and 14 days**

Note: if using patches, on how many days have you been wearing a patch?

☐

Q7 Over the past **month**, how helpful have you found opioid pain medicines in relieving your pain? **Circle ONE answer only**

Not at all helpful	A little helpful	Moderately helpful	Very helpful	Extremely helpful

Q8 Over the past **month**, have opioid pain medicines relieved your pain as much as you expected? **Circle ONE answer only**

Definitely yes	Probably yes	Might or might not	Probably not	Definitely not

Part 2: About your experiences with opioid pain medicines

The following questions are about your experiences with opioid pain medicines over the whole time that you have been using them.

Q9 There have been times when opioid pain medicines have caused me to lose interest in my usual activities. **Circle ONE only**

Strongly disagree	Disagree	Neutral	Agree	Strongly agree

Q10 There have been times when opioid pain medicines have caused me to have trouble concentrating or remembering. **Circle ONE only**

Strongly disagree	Disagree	Neutral	Agree	Strongly agree

Q11 There have been times when opioid pain medicines have caused me to feel slowed down, sluggish or sedated. **Circle ONE only**

Strongly disagree	Disagree	Neutral	Agree	Strongly agree

Q12 There have been times when opioid pain medicines have caused me to feel depressed, down or anxious. **Circle ONE only**

Strongly disagree	Disagree	Neutral	Agree	Strongly agree

Q13 There have been times when the side effects of opioid pain medicines have interfered with my work, family, or social responsibilities? **Circle ONE only**

Strongly disagree	Disagree	Neutral	Agree	Strongly agree

Q14 There have been times when opioid pain medicines have made it hard for me to think clearly? **Circle ONE only**

Strongly disagree	Disagree	Neutral	Agree	Strongly agree

Q15 There have been times when opioid pain medicines have made me sleepy or less alert when I was doing something where I needed to be alert? **Circle ONE only**

Strongly disagree	Disagree	Neutral	Agree	Strongly agree

Q16 Considering any side effects of opioid pain medicines you may have experienced, how bothersome were these side effects? **Circle ONE only**

No side effects	A little bothersome	Moderately bothersome	Very bothersome	Extremely bothersome

Q17 There have been times when I have been preoccupied with or thought constantly about my opioid pain medicines. **Circle ONE only**

Strongly disagree	Disagree	Neutral	Agree	Strongly agree

Q18 There have been times when I have felt that I could not control how much or how often I needed opioid pain medicines. **Circle ONE only**

Strongly disagree	Disagree	Neutral	Agree	Strongly agree

Q19 There have been times when I have needed to increase the dose of my opioid pain medicines to get the same effect. **Circle ONE only**

Strongly disagree	Disagree	Neutral	Agree	Strongly agree

Q20 There have been times when I have worried that I might be dependent on or addicted to opioid pain medicines. **Circle ONE only**

Strongly disagree	Disagree	Neutral	Agree	Strongly agree

Q21 There have been times when I have wanted to stop my opioid pain medicines or to cut down on the amount that I use. **Circle ONE only**

Strongly disagree	Disagree	Neutral	Agree	Strongly agree

Q22 There have been times when opioid pain medicines have caused me to have problems with family, friends or co-workers. **Circle ONE only**

Strongly disagree	Disagree	Neutral	Agree	Strongly agree

Q23 There have been times when family or friends have thought that I may be dependent on or addicted to opioid pain medicines. **Circle ONE only**

Strongly disagree	Disagree	Neutral	Agree	Strongly agree

Part 3: About your opioid pain medicine prescription, information and support

Q24 Who usually supplies your pain medicine prescription? **Tick ONE only**

- A local community pharmacy ☐ Go to Q25
- An online pharmacy ☐ Go to Q27
- A dispensing doctors surgery ☐ Go to Q27

Note: *Dispensing doctors are GP practices who supply prescription medicines to patients when there are no local community pharmacies. They are not community pharmacies located within GP practices.*

Q25 Who usually collects your pain medicine prescription? **Tick ONE only**

- I collect the prescription myself ☐
- Someone else collects the prescription for me ☐
- My pharmacy delivers the prescription for me ☐

Q26 Approximately how far do you have to travel to your local community pharmacy? **Tick ONE only**

- Up to 1 mile ☐
- Between 1 and 2 miles ☐
- Between 2 and 5 miles ☐
- More than 5 miles ☐
- Don't know ☐

Q27 Have you ever spoken to a local community pharmacist about a **prescription** medicine in any of the following ways? **Tick ALL that apply**

Note: This includes prescription medicines for any condition, not just pain, but does not include medicines you have bought

- Asked for advice at the pharmacy counter ☐
- Made a telephone call to the pharmacy for advice ☐
- Had a telephone or in-person appointment with a pharmacist about a new medicine (the New Medicine Service) ☐
- Had a review of any long-term medicines with a pharmacist (a Medicine Use Review) ☐
- None of the above ☐

Q28 During the time you have been using opioid pain medicines, can you remember having any of the following information from a healthcare professional? (e.g. doctor, nurse, pharmacist) **Tick ALL that apply**

- How to use the medicine ☐
- How long you were likely to be using the medicine for ☐
- How effective the medicine was likely to be in treating your pain ☐
- The common side effects e.g. constipation, drowsiness ☐
- Information about driving when taking the medicine ☐
- The medicine (or current dose) may not give the same amount of pain relief if it is taken for a long time ☐
- Missing a dose or reducing the dose can make people feel unwell if the medicine has been taken for a long time ☐
- Not to take any other medicines that contain opioids which you can buy from a pharmacy ☐
- The effects of drinking alcohol with the medicine ☐
- Storing the medicine in a safe place ☐
- Returning any unused medicines to the pharmacy ☐
- Not to share your pain medicines with others ☐
- None of the above ☐
- Can't remember ☐

Q29 Do you think having more information about opioid pain medicines at the **start** of treatment would have been useful to you? **Circle ONE only**

Definitely yes Probably yes Might or might not Probably not Definitely not

Q30 When receiving information about a medicine or health condition from a healthcare professional, what type of information works best for you? **If you prefer to receive information in more than one way, tick ALL that apply**

- Someone talking to you ☐
- Having a leaflet to take home ☐
- Looking at the information on a website ☐
- Watching an online video ☐

Q31 Are you happy to have information about a medicine or health condition from a community pharmacist? **Circle ONE only**

Definitely yes	Probably yes	Might or might not	Probably not	Definitely not

Q32 Local community pharmacists already provide a follow up service for some new medicines. The service supports people to use new medicines in the most effective way, manage any side effects and checks the medicine is working. Advice is given when needed and patients can be referred back to their GP if there are problems.

Do you think this type of service could be useful for opioid pain medicines? **Circle ONE only**

Definitely yes	Probably yes	Might or might not	Probably not	Definitely not

Part 4: About you

Q33 What is your age? **Enter your age in years in the box**

[ ]

Q34 What is your gender?

- Male ☐
- Female ☐
- Non-binary ☐
- Transgender ☐
- Intersex ☐
- Prefer not to say ☐

Q35 What is your ethnic group?

White	Mixed Heritage	Asian
White British ☐	White and Black Caribbean ☐	Indian ☐
White Irish ☐	White and Black African ☐	Pakistani ☐
White Scottish ☐	White and Asian ☐	Bangladeshi ☐
White Welsh ☐	Mixed Other (please specify)	Asian Other (please specify)
White Other (please specify)	....	....
....		
**Black or Black British**	**Chinese or any other group**	
Caribbean ☐	Chinese ☐	
African ☐	Any Other (please specify)	
Black Other (please specify)	....	
....		

Q36 Employment details: are you currently?

- Employed ☐
- A student ☐
- Retired ☐
- A homemaker ☐
- Unable to work ☐
- Out of work and seeking opportunities ☐

Q37 Has your employment situation changed due to the coronavirus pandemic?

- Yes ☐
- No ☐

Table 1 Associations between PEG scale components and how helpful respondents found opioids in relieving pain over the past month

Scale component [range 0-10]	Not at all / A little / Moderately helpful, n (%)		Very / Extremely Helpful, n (%)		p-value [Chi Square]
P score (n = 614)					
Low [0-3]	12	(44.4)	15	(55.6)	<0.001*
Medium [4-6]	92	(49.2)	95	(50.8)	
High [7-10]	258	(64.5)	142	(35.5)	
E score (n = 612)					
Low [0-3]	26	(44.8)	32	(55.2)	0.037
Medium [4-6]	73	(56.2)	57	(43.8)	
High [7-10]	262	(61.8)	162	(38.2)	
G score (n = 613)					
Low [0-3]	23	(44.2)	29	(55.8)	0.013
Medium [4-6]	80	(53.7)	69	(46.3)	
High [7-10]	258	(62.6)	154	(37.4)	

*Additional associations tested after original significance set to p<0.01. If included in original analysis, significant p-value would be reduced to p<0.006 via Bonferroni adjustment.

PEG, Pain intensity, interference with Enjoyment, interference with General activity.
