# Supplementary material for: Difficulties with prescribed opioids: a cross-sectional survey of primary care patients in England, United Kingdom
Source: Pain Rep. 2025 Feb 25;10(2):e1246. doi: 10.1097/PR9.0000000000001246 (PMC11864306; doi:10.1097/PR9.0000000000001246)
Supplement: SUPPLEMENTARY MATERIAL [file painreports-10-e1246-s002.pdf]

Associations between PEG scale components and how helpful they found opioids in relieving pain over the past month

Scale component [range 0-10]	Not at all / A little / Moderately helpful, n (%)		Very / Extremely Helpful, n (%)		p-value [Chi Square]
P score (n = 614)					
Low [0-3]	12	(44.4)	15	(55.6)	<0.001*
Medium [4-6]	92	(49.2)	95	(50.8)	
High [7-10]	258	(64.5)	142	(35.5)	
E score (n = 612)					
Low [0-3]	26	(44.8)	32	(55.2)	0.037
Medium [4-6]	73	(56.2)	57	(43.8)	
High [7-10]	262	(61.8)	162	(38.2)	
G score (n = 613)					
Low [0-3]	23	(44.2)	29	(55.8)	0.013
Medium [4-6]	80	(53.7)	69	(46.3)	
High [7-10]	258	(62.6)	154	(37.4)	

*Additional associations tested after original significance set to p<0.01. If included in original analysis, significant p-value would be reduced to p<0.006 via Bonferroni adjustment.

PEG, Pain intensity, interference with Enjoyment, interference with General activity.
